# Stability of mesoporous silica using ricinoleic methyl ester as a template with the addition of HCl and application of Cd^2+^ adsorption optimized by Box–Behnken design

**DOI:** 10.1039/d2ra06973c

**Published:** 2023-03-06

**Authors:** Andriayani, Marpongahtun, Yugia Muis, Jessica Pakpahan, Amru Daulay

**Affiliations:** a Department of Chemistry, Faculty of Mathematics and Natural Sciences, Universitas Sumatera Utara Jl. Bioteknologi No. 1 Medan 20155 Indonesia andriayani@usu.ac.id; b Graduate School, Department of Chemistry, Faculty of Mathematics and Natural Sciences, Universitas Sumatera Utara Jl. Bioteknologi No. 1 Medan 20155 Indonesia; c Postgraduate School, Department of Chemistry, Faculty of Mathematics and Natural Sciences, Universitas Sumatera Utara Jl. Bioteknologi No. 1 Medan 20155 Indonesia

## Abstract

Mesoporous silica is restricted to organic solvents or other acidic media. The application of mesoporous silica depends on the medium's chemical stability and mechanical properties. It is necessary to stabilize the mesoporous silica material under acidic conditions. The results of the nitrogen adsorption characterization show that MS-50 has a large surface area and porosity, resulting in good mesoporous silica. Using variance analysis (ANOVA) to compare the collected data, the best conditions were found at a pH of 6.32, a Cd^2+^ concentration of 25.30 ppm, an adsorbent dose of 0.06 g, and a time of 70.44 min. The Cd^2+^ adsorption experiment data best fit the Langmuir isotherm model with the maximum amount of Cd^2+^ that MS-50 could absorb being 103.10 mg g^−1^.

## Abstract 2

Illustration of the adsorption of Cd^2+^ by mesoporous silica.

## Graphical abstract

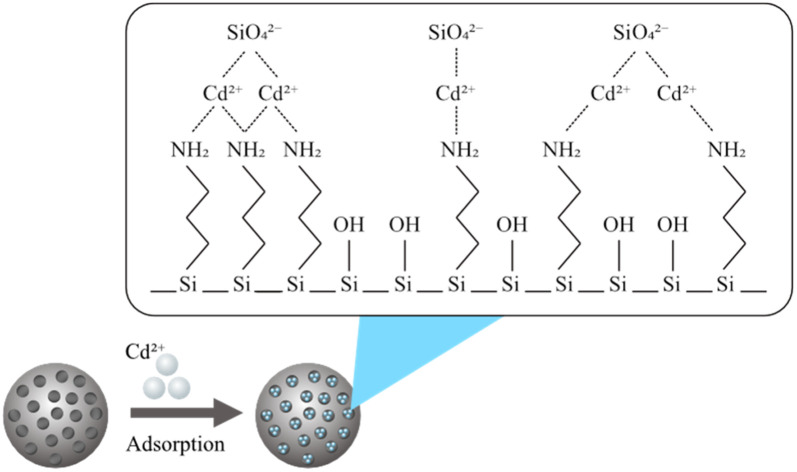

## 1. Introduction

Mesoporous silica is used in many applications, such as catalysis,^1^ drug delivery,^2^ sensing,^3^ and optics.^4^ It is also used in ion separation,^5^ environmental applications,^6^ metallurgy,^7^ and nuclear applications.^8^ The studied mesopores are ligands that extract heavy metals from waste fluxes.^9^

Mesoporous silica synthesis can be used in two pathways: forming the cooperative self-assembly and liquid-crystal template processes.^10^ This synthesis process is based on the interaction between organic templates (surfactants in the liquid crystal structure and inorganic precursors) such as silica, which forms stable mesoporous silica.^11^ Applying high temperatures and chemicals to the template produces mesoporous silica with a small, well-organized pore structure. The obtained mesoporous silica has a high surface area, which is excellent for various applications. However, mesoporous silica has fragile pores that are not durable.^12^

Heavy metal pollution has become a major environmental problem threatening the environment and public health. The heavy metals pollute the soil, water, and air. It also can quickly get into the food chain and cause long-term, toxic effects on living organisms. One of the adsorption method that have been used to remove Cd^2+^ effectively is because it is simple, effective, and cheap.^13^ Cd^2+^ in water can be removed with adsorption materials, such as activated carbon^14^ and clays.^15^ However, these materials are not good at removing Cd^2+^ because they have low pore volume and a poor pore structure.

The porosity of a material affects its physical qualities, such as density, the conductivity of heat, and strength, due to its regular pore structure, high surface area, and consistent pore size distribution.^16^ Hybrid materials are formed when mesoporous silica materials are combined with organosilane due to its broad applicability in various disciplines. Inorganic and organic building components are dispersed on the nanoscale in hybrid materials, and serve as an adsorbent.^17^

Response surface methodology (RSM) is a method that looks at the relationship between some independent factors and some dependent responses.^18^ The most common designs in RSM are the Central-Composite Design (CCD) and the Box–Behnken design (BBD).^19^ In RSM, BBD is thought to be an efficient option. In different studies, BBD has been used to find the best conditions for the desired results.^20^ BBD was used to study the simultaneous effects of the independent variables on the removal of Cd^2+^ (as the dependent variable) to find the optimum conditions for Cd^2+^ removal from aqueous solutions.

Mesoporous silica is restricted to organic solvents or other acidic media. However, synthesis of mesoporous silica in ion separation (in environmental or hydrometallurgical ore separation processes), the treats are often acidic or even very acidic. The application of mesoporous silica in this field highly depends on the medium's chemical stability and mechanical properties.^21^ In previous studies, mesoporous silica was synthesized using ricinoleic methyl ester as a template. This study synthesized mesoporous silica with variation methanol to obtain the best mesoporous silica. This study showed that methanol at 18 g and HCl at 45 mL were able to obtain mesoporous silica with a BET surface area of 163.71 m^2^ g^−1^, a pore size of 12 nm, and was also able to adsorb Cu^2+^ with a removal percentage of 82.36%.^22^ In this study, synthesized mesoporous silica using an organic solvent, ricinoleic methyl ester, as a template was carried out by adding 18 g of methanol and varying HCl volumes of 20 mL, 30 mL, 40 mL, and 50 mL to increase the mesoporous silica's stability and application with adsorption of Cd^2+^ optimized by the Box–Behnken design.

## 2. Experimental section

### 2.1. Materials

Hydrochloric acid (HCl 37 wt%), 3-aminopropyltrimethoxysilane (APMS), methanol (CH_3_OH 98 wt%), and tetraethylorthosilicate (TEOS, 98%) were purchased from Sigma-Aldrich, and deionized water (DI) was obtained from PT Sumber Aneka Karya Abadi. Methyl ester ricinoleic acid (C_19_H_36_O_3_) was obtained from *Ricinus communis*.

### 2.2. Synthesis mesoporous silica

In two neck flasks, 0.0145 mol of methyl ester ricinoleic acid was added to 100 mL of DI water, stirring for 30 min (mixture *A*). Then, a mixture of 0.0058 mol APMS, 0.028 mL of TEOS, 18 g of methanol, and *x* mL HCl (*x* = 20 mL, 30 mL, 40 mL, and 50 mL), was stirred for 10 min (mixture *B*). Then, mixture *A* and mixture *B* were stirred for 2 h. Then, the mixtures were heated in an oven at 80 °C for 72 h to form a porous solid. The product was separated and dried at 50 °C. The resulting solid was calcined at 550 °C for 6 h to remove the organic impurity. Five samples were named MS-20, MS-30, MS-40, and MS-50, where the numbers 20, 30, 40, and 50 refer to the volume of HCl.

### 2.3. Characterization

Functional groups were characterized using Fourier-transform infrared spectroscopy (FTIR) (Shimadzu) with a KBr pellet technique. The porous material was characterized by adsorption–desorption at a temperature of 77 K using a Quantachrome instrument. Prior to characterization, samples were evacuated at 350 °C for 4 h. Nitrogen adsorption was used to measure: (i) the specific surface area using the Brunauer–Emmet–Teller (BET) method; (ii) the surface area was calculated using the *t*-plot method by subtracting the surface area of BET with the surface area, and the outer surface could be identified; and (iii) the pore size using the Barret–Joyner and Halenda (BJH) method was accurate with a cylindrical structure and cubic structure. Phase analysis of the material by X-ray diffraction (XRD) was recorded with Cu Kα (*λ* = 0.15406 nm) using a 2*θ* range of 10°–90°. The morphology was investigated by scanning electron microscope (SEM) using SEM JED-2300, Jeol.

### 2.4. Optimization of the adsorption process

The current work is focused on a four-level Box–Behnken design (BBD) in response surface methodology (RSM) to optimize the % removal of Cd^2+^, solution pH, adsorbent dose, concentration, and times. Statistical analysis software [Design expert software version 13] was used to determine how important every single factor, interaction, and quadratic term was in the optimization process. Table 1 shows the factorial design.

**Table:** Factors and their levels of BBD

Factor	Levels			Description
	High (+1)	Central (0)	Low (−1)	
*A*	2	5.5	9	*A*: pH
*B*	0.05	0.1	0.15	*B*: dose (g)
*C*	20	50	80	*C*: concentration (ppm)
*D*	30	60	90	*D*: time (min)

In this study, 27 experiments were conducted to determine how the four main independent factors affected the efficiency and effectiveness of the removal. A non-linear regression method was used to fit the second-order polynomial to the experimental data and determine the important model terms.

The design included 27 experiments to find the best independent factors levels. This study investigated four factors in the laboratory. Each was chosen as an independent variable with three levels: pH (*A*, 2–9), adsorbent dose (*B*, 0.05–0.15 g), Cd^2+^ ions concentration (*C*, 20–80 ppm), and contact time (*D*, 30–90 min). The parameter in the regression equation was studied, and variance analysis (ANOVA) was used to find most important parameters in the model. Mesoporous silica was used for the adsorption experiments under different process conditions, such as pH, adsorbent dose, process time, and concentration of Cd^2+^. The concentrations of Cd^2+^ were determined before and after adsorption using atomic absorption spectroscopy (AAS, iCE 3300). Once the Cd^2+^ concentration was known, eqn (1) was used to estimate the equilibrium amount adsorbed (mg g^−1^), and eqn (2) was used to estimate the removal percentage (%) calculated as,1
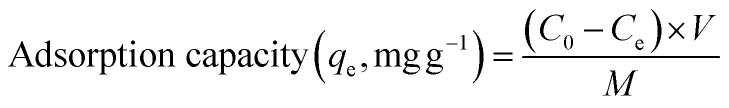
2
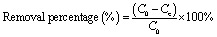


**Fig. 1:**
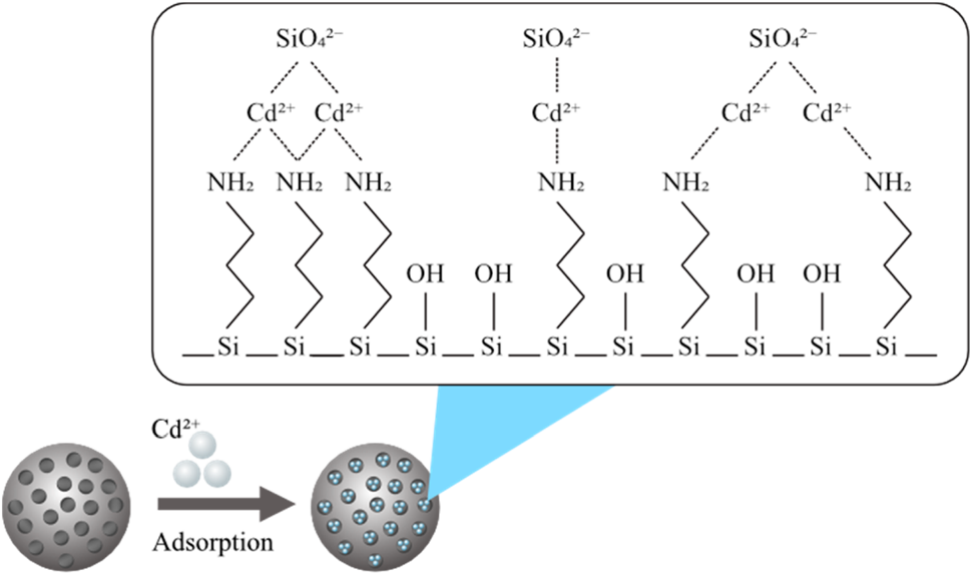
Illustration of Cd^2+^ adsorption by mesoporous silica.^22^

In the above equations, *C*_0_ (ppm) is the initial concentration of Cd^2+^, and *C*_e_ (ppm) is the final concentration of Cd^2+^. The adsorption capacity (*q*_e_) is the amount of Cd^2+^ adsorbed per unit mass of the mesoporous silica (mg g^−1^). *V* is the volume of the solution (L), and *M* is the mass of the mesoporous silica (g). The schematic illustration of mesoporous silica and Cd^2+^ adsorption can be seen in Fig. 1. The actual BBD experimental design matrix is shown in Table 2.

**Table:** The 4-factors BBD matrix and experimental data for Cd^2+^ removal efficiency

Run	pH	Dose (g)	Concentration (ppm)	Time (min)	Removal predicted (%)	Removal actual (%)
1	10	0.15	50	60	83.61	82.42
2	5.5	0.15	50	30	75.02	75.17
3	10	0.05	50	60	81.00	79.82
4	5.5	0.1	50	60	83.26	82.91
5	5.5	0.15	50	90	87.37	88.25
6	5.5	0.1	50	60	83.26	84.92
7	5.5	0.1	20	90	90.09	89.82
8	5.5	0.1	50	60	83.26	81.96
9	5.5	0.15	20	60	76.81	76.85
10	5.5	0.1	20	30	76.94	76.82
11	5.5	0.05	80	60	85.91	85.73
12	5.5	0.1	80	90	94.79	93.91
13	1	0.1	20	60	78.83	78.89
14	1	0.1	50	30	80.10	79.82
15	10	0.1	20	60	85.21	85.64
16	1	0.15	50	60	75.78	75.96
17	5.5	0.15	80	60	84.48	84.45
18	5.5	0.05	50	30	78.63	78.92
19	10	0.1	50	30	77.23	77.95
20	5.5	0.05	20	60	83.55	83.44
21	1	0.05	50	60	86.56	86.75
22	1	0.1	50	90	88.93	88.08
23	5.5	0.1	80	30	82.28	81.55
24	10	0.1	80	60	84.97	86.08
25	10	0.1	50	90	94.06	94.21
26	1	0.1	80	60	89.10	89.84
27	5.5	0.05	50	90	91.93	92.95

## 3. Results and discussions

Mesoporous silica was synthesized using ricinoleic methyl ester as a template with the addition of varying amounts of HCl. Ricinoleic methyl was used as a template based on the literature.^16,22^ The seed *Ricinus communis* extracts castor oil.^23^ Castor oil is unusual among vegetable oils because it is the sole source of hydroxylated fatty acids.^24^ Castor oil is extracted from the castor plant by pressing or solvent extraction, forming methyl ester when vegetable oils or animal fats combine with alcohol in a chemical reaction.^25^ Methanol and ethanol are the most used alcohols. Ricinoleic methyl ester in its carbon chain has a double bond, a hydroxyl group, and a carboxylate group that can affect the formation of micellar aggregates. It can affect the porous properties of the obtained mesoporous silica material. Some conditions that can affect the regularity of the pores include pH, the ratio of the components of the reaction mixture, solvent, and temperature. The degree of surfactant ionization, which is the ratio of the molar charge to the total surfactant, can change with the addition of acid because the pH value will affect the charge density of micelles and can result in different template arrangements.^26^ The addition of acid can also affect the hydrolysis and condensation reactions of the silicate material because the addition of acid causes the oxygen atom of the silanol group and the siloxy group to be protonated rapidly, and release alcohol and water.^27^ Other reaction conditions, such as timing the addition of silicate components (for example, by delaying the addition of TEOS), will also be carried out. By timing the addition, it will give time for interaction between the amine group of APMS and the charge of the head group of the template, which can affect the formation of pores. Nikolic *et al.* researched silica synthesis's effect and the silicate's mixing time with TEOS. The results show that the right time can produce the best silica. In this study, the time obtained was around 7 min.^28^ Likewise, Alfawaz *et al.* studied the right time to add alcohol to the synthesis of mesoporous silica. The correct time was around 20 min.^29^ Another factor that can affect the formation of mesostructured pores is the addition of alcohol since alcohol can function as a co-solvent, increasing the reaction mixture's polarity.^30^ The synthesis of mesoporous silica with the addition of HCl can change the density of the template ricinoleic methyl ester and APMS. Adding HCl can release methyl ions from ricinoleic methyl ester to COO^−^, while protonating the amine group from APMS to NH_3_^+^. There is then an effective interaction between COO^−^ with NH_3_^+^, which will determine the nature of the pores in the silica material.

**Fig. 2:**
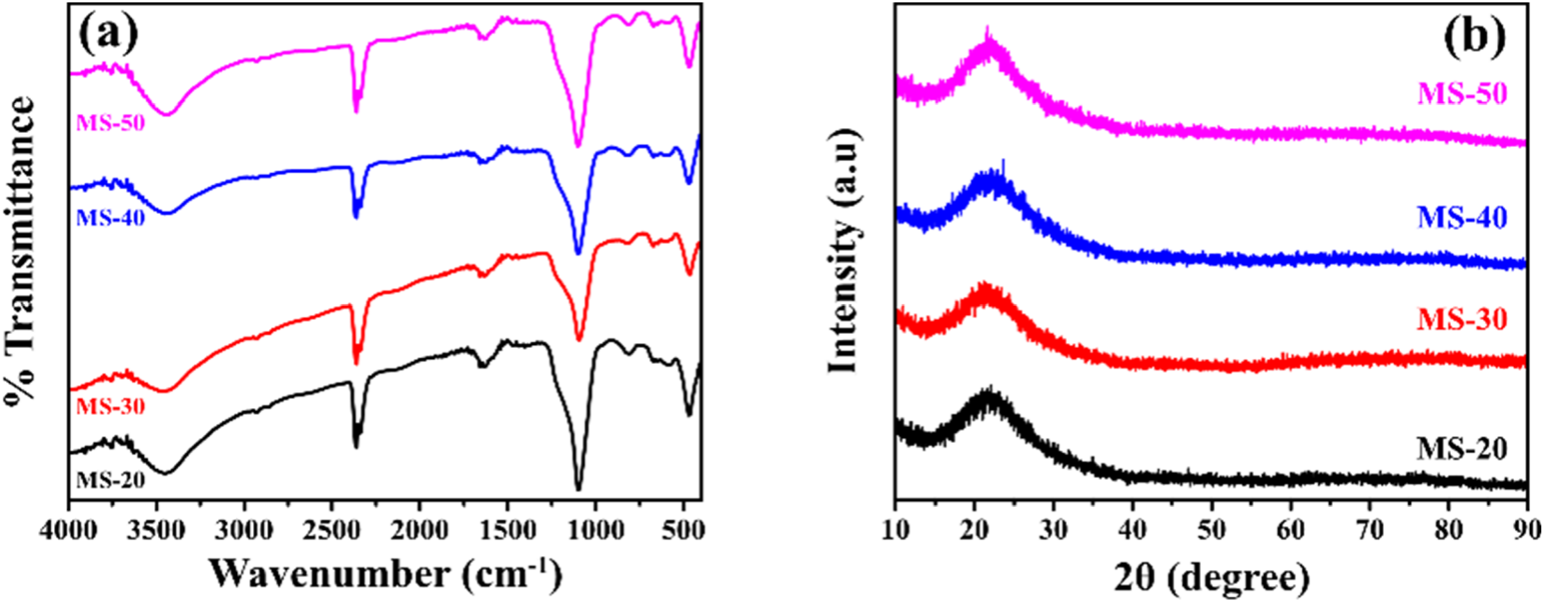
(a) FTIR spectra of mesoporous silica, (b) diffractogram XRD of mesoporous silica.

**Fig. 3:**
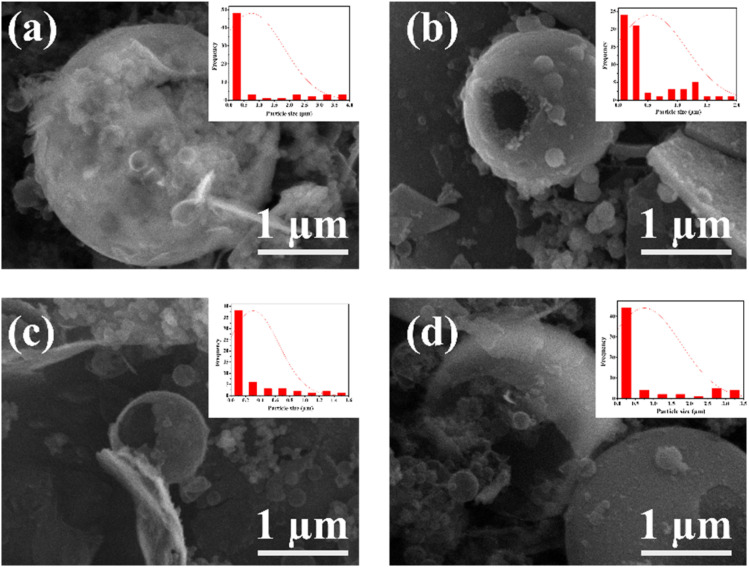
SEM images of (a) MS-20, (b) MS-30, (c) MS-40, and (d) MS-50 with magnification of 200 00×.

The FTIR spectra can be seen in Fig. 2a. All products of mesoporous silica showed an absorption peak between 3433 cm^−1^ to 3447 cm^−1^ that was widened due to OH (Si–OH).^31^ The other absorption peaks at 1089 cm^−1^ to 1118 cm^−1^ are strong due to the asymmetric stretching of Si–O–Si (*υ*_as_ Si–O–Si),^32^ and the features at 808 cm^−1^ to 836 cm^−1^ are due to the presence of symmetrical groups Si–O–Si (*υ*_is_ Si–O–Si).^33^ The features in the wavenumber range of 456–471 cm^−1^ is due to Si–O. In addition to the characteristic H–OH water twisting band at 1600 cm^−1^ and 2400 cm^−1^.^34^ The functional group has no significantly different FTIR spectral features from MS-20 to MS-50. The frequency of the characteristic peak of Si–O changed when Cd was added. The covalent radius of Si is 116 pm and the covalent radius of cadmium is 148 pm. If Cd ions were to enter the Si–O tetrahedron, the vibration would be harder because the covalent radius of Cd is larger than that of Si. It showed that Cd^2+^ could bind with Si–O, and proved that silicic acid or polysilicic acid and Cd could form a water–soluble complex.^35^ The XRD diffractogram can be seen in Fig. 2b at an angle of 2*θ* between 10° to 30°, showing that all shapes are equal to the broad peak (broad), and the diffractogram peak is at 24.0°, which indicates that the mesoporous silica is amorphous.^36,37^ There is no significant difference in the XRD diffractograms of MS-20 to MS-50. SEM images of MS-20 (Fig. 3a), MS-30 (Fig. 3b), and MS-40 (Fig. 3c) showed a particle shape in the form of a mixture consisting of dispersed round plate particles (such as coins) of small size, and some formed agglomerations. Other particles in the form of spherical spheres,^38,39^ which are dispersed with a larger size, have thin skin so that they are easily broken. MS-20 also has plate particles in thicker and irregular shapes. MS-30 and MS-40, with a larger spherical shape, has a better shape. Some particles have damage to their skin, resulting in a hole pore. This condition shows that spherical particles with a larger size have a thicker plate. The skin thickness of the spherical particles is thicker than the size of the skin in the particles of MS-20. MS-40 has a spherical particle shape with a larger size, and has a thin skin so that it is easily damaged, and there are particles in the form of a thicker plate with a large size. MS-50 (Fig. 3d) has particles that are dominated by small round plate particles, some of which are dispersed and some forming agglomerations. Other particles are in the form of spherical shapes with a larger size and have thin skin that is easily damaged. Particle sizes for MS-20, MS-30, MS-40, and MS-50 were dominated by 0.77 μm, 0.65 μm, 0.51 μm, and 0.49 μm, respectively. It shows the size of the particles in the form of micropores. Furthermore, it shows that upon the increasing addition of HCl, smaller particle sizes were obtained. Based on the amorphous XRD diffractogram and spherical SEM images, the resulting mesoporous silica is similar to SBA 15, as it is known that the SBA 15 diffractogram is amorphous^40^ and the SEM images are spherical.^41^

**Fig. 4:**
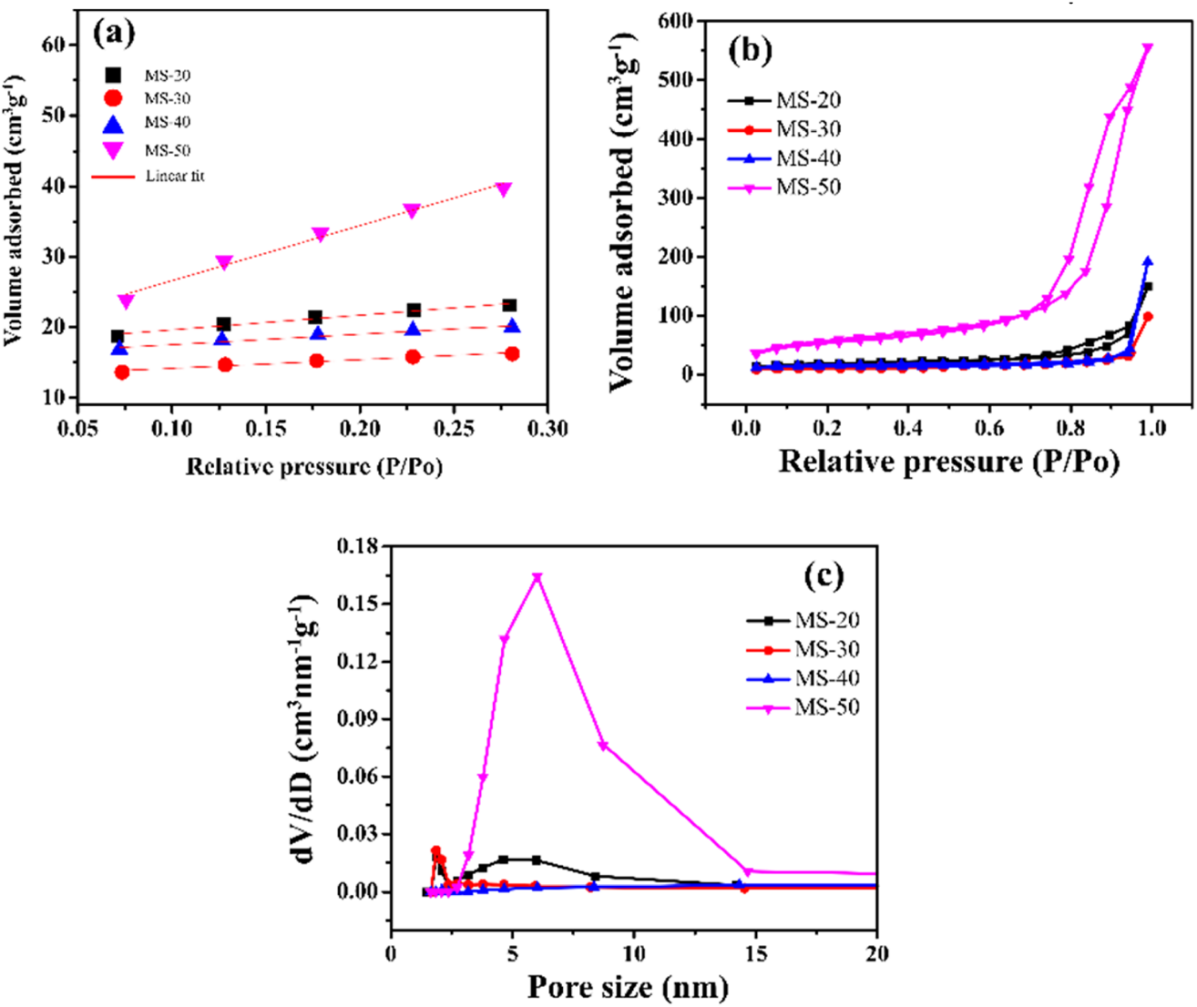
N_2_ adsorption by mesoporous silica: (a) *t*-plot, (b) Brunauer–Emmett–Teller (BET) and (c) Barrett–Joyner–Halenda (BJH).

N_2_ adsorption characterization using *t*-plot, BET surface area and BJH pore volume can be seen in Fig. 4. The *t*-plot from BET in Fig. 4a shows the *R*^2^ of MS-20, MS-30, MS-40, and MS-50 at up to 0.99. The BET surface area in Fig. 4b shows hysteresis at high relative pressure values with large pores. The BET surface area of MS-20 was 46.23 m^2^ g^−1^, whereas that of MS-30 was 24.30 m^2^ g^−1^, MS-40 was 16.55 m^2^ g^−1^, and MS-50 was 250.78 m^2^ g^−1^. The BJH pore volume is presented in Fig. 4c. The results of the pore sizes of MS-40 and MS-50 are 14.32 nm and 6.01 nm, respectively, and can be called mesoporous silica. Ijaz *et al.* also conducted experiments regarding the synthesis of mesoporous silica with varying amounts of HCl. If HCl is given in small amounts, it produces few pores and forms a large surface area. However, if HCl is given in large quantities, it will give a large pore, but a small surface area. The appropriate addition of HCl will provide a large surface area coupled with a large position, making it very suitable to form mesoporous silica.^42^ Oo *et al.* performed experiments on synthesizing mesoporous silica with the addition of HCl with the optimization of the RSM method. It was found that the administration of HCl impacted the resulting surface area and pore size. Applying HCl in the right amount can produce mesoporous silica material with optimal surface area and pore size.^43^ Zhang *et al.* investigated the stability of mesoporous silica with varying amounts of lauric acid. It was found that producing mesoporous silica with high porosity and large surface area requires the right amount of lauric acid. The addition of too little lauric acid will result in small pores. If too much is added, it will result in large pores.^44^ In the end, this research focused on variations in pH, especially the addition of HCl in the synthesis of mesoporous silica. The addition of the right pH conditions will produce mesoporous silica with high porosity and a large surface area. However, solvent, temperature, and cooling time variations were not carried out. Zhou *et al.* performed the synthesis of mesoporous silica with temperature variations. The result is that the higher the temperature, the higher the pore will be obtained.^45^ Lee *et al.* synthesized mesoporous silica with various solvents. It was found that an acidic solvent will increase the pore and surface area, but a slightly alkaline solvent will shrink the surface area.^46^ Kang *et al.* performed the synthesis of mesoporous silica with cooling time. It was shown that the longer the cooling time, the larger the surface area and the larger the pores (Table 3).^47^

**Table:** Physical properties of mesoporous silica

Samples	BET surface area (m^2^ g^−1^)	Pore volume (cm^3^ g^−1^)	Pore size (nm)
MS-20	46.23	0.22	1.86
MS-30	24.30	0.14	1.85
MS-40	16.55	0.28	14.32
MS-50	250.78	0.90	6.01

**Fig. 5:**
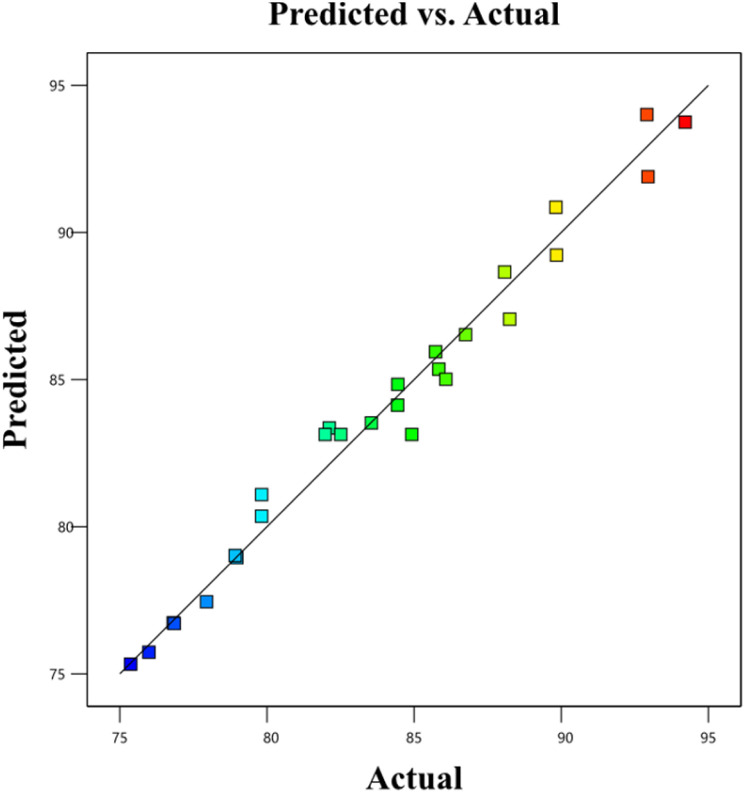
The plot of the relationship between the predicted and actual values of Cd^2+^ removal (%).

**Fig. 6:**
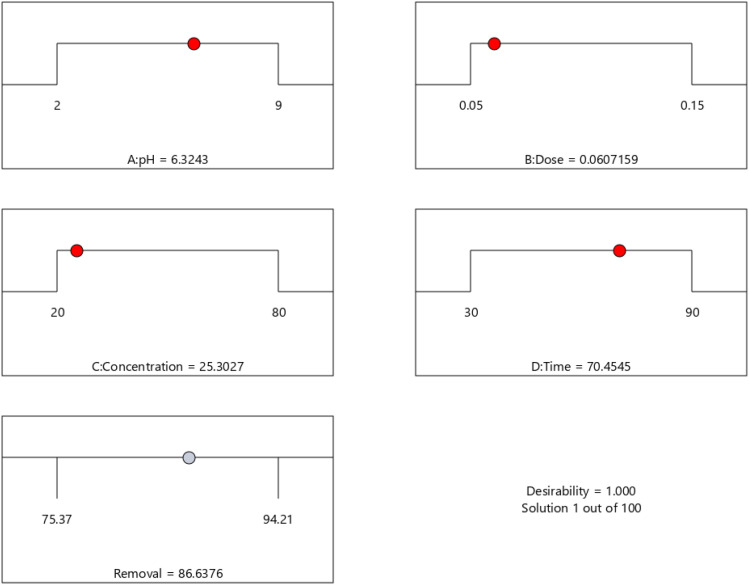
The optimum conditions for the removal of Cd^2+^ using MS-50.

**Fig. 7:**
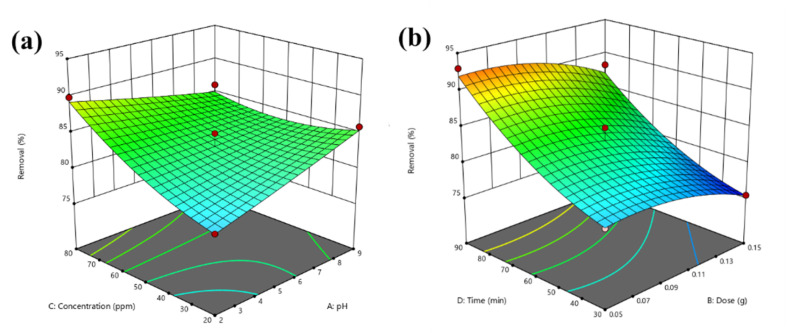
Response surfaces for the BBD of (a) pH – Cd^2+^ concentration and (b) adsorbent dosage – time.

BBD planned a total of 27 experiments (Table 2). For the four factors that were examined, pH (*A*), adsorbent dose (*B*), Cd^2+^ ions concentration (*C*), and contact time (*D*) were studied as independent process factors, and their effects on the Cd^2+^ removal efficiency (the response) were studied using the BBD approach. The mathematical relationship between the response and the process factors was figured out using a quadratic polynomial model. The regression model for the response was tested for significance, and the results of ANOVA tests are shown in Table 4. Values of model terms Prob > *F* < 0.05 mean that factors are significant under certain conditions. Significant model terms for the response (Cd^2+^ removal) are *A*, *B*, *C*, *A*^2^, *C*^2^, *AB*, *AC*, and *BC*. It was found that pH (*A*) does not significantly affect the percentage of the Cd^2+^ removal efficiency. It is probably because the adsorption process is not as sensitive to changes in pH. ANOVA for response factors shows that the value of *R*-squared (determination coefficient) is 0.97, which is very high and shows a good relationship between the actual and predicted values, as shown in Fig. 5. The optimum conditions for the MS-50 to Cd^2+^ removal are effectively presented in Fig. 6. The calculations were done. The desirability value for Cd^2+^ showed that the maximum adsorption efficiency was 86.63% at the optimum conditions: contact time of 70.44 min, pH of 6.32, a dose of 0.06 g, and concentration of Cd^2+^ of 25.30 ppm. In the experiments, the initial pH and concentration of Cd^2+^ were changed to show how well Cd^2+^ was removed. The results showed that the pH of the solution has an essential effect on how well the process works (Fig. 7). The efficiency of Cd^2+^ adsorption decreases as the pH increases. This is caused by changes in the surface charges of the adsorbent and Cd^2+^ as a function of the pH. One of the most important things to consider is how the concentration of the ions at the beginning affects the process. With the initial concentration of the removal percentage, Cd^2+^ decreases. This can be explained by the fact that there are a limited number of active sites on the surface of the adsorbent, which are filled up after a particular optimum concentration.^48^ It is expected that this would happen because of a significant force. It happens because the concentration and solution of Cd^2+^ on the surface of the adsorbent are more significant when the initial concentration of Cd^2+^ is higher, and the amount of adsorbent is the same.^49^

**Table:** ANOVA of the response surface quadratic model for Cd^2+^ removal efficiency

Source	Sum of squares	*F*-value	*p*-value	
Model	743.33	39.42	<0.0001	Significant
*A* – pH	3.59	2.66	0.1287	
*B* – adsorbent	54.53	40.49	<0.0001	
*C* – concentration	74.05	54.99	<0.0001	
*D* – time	453.75	336.92	<0.0001	
*AB*	42.64	31.66	0.0001	
*AC*	28.20	20.94	0.0006	
*AD*	16.00	11.88	0.0048	
*BC*	9.95	7.39	0.0187	
*BD*	0.3306	0.2455	0.6292	
*CD*	3.31	2.46	0.1428	
*A* ^2^	0.0984	0.0731	0.7915	
*B* ^2^	13.55	10.06	0.0080	
*C* ^2^	9.93	7.37	0.0188	
*D* ^2^	16.94	12.58	0.0040	
Residual	16.16			
Lack of fit	11.25	0.4582	0.8365	Not significant
Pure error	4.91	39.42		
Cor. total	759.49			

**Fig. 8:**
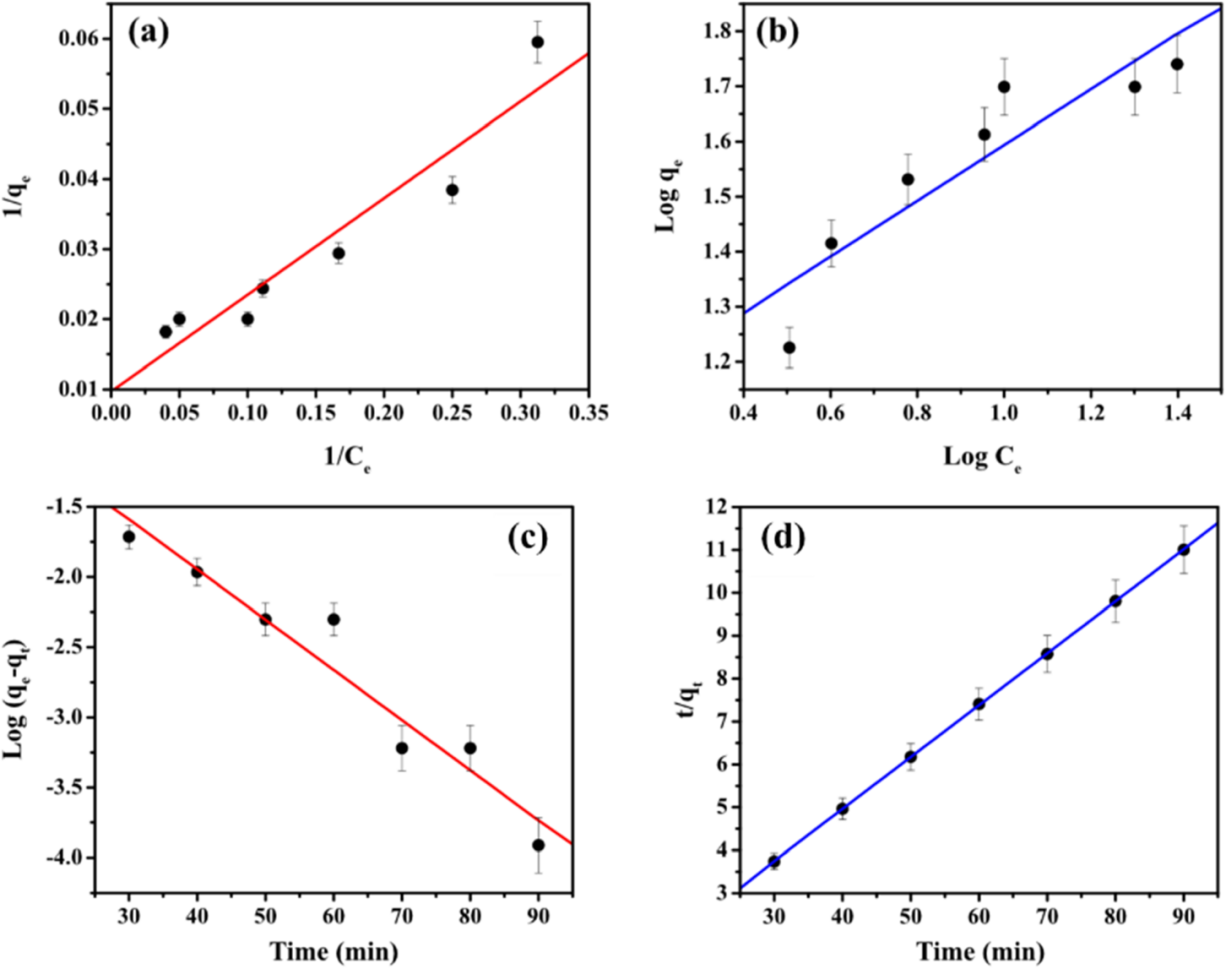
The adsorption of Cd^2+^ onto MS-50 of the (a) Langmuir and (b) Freundlich isotherm model. Kinetics plots for the removal of Cd^2+^ by MS-50 of (c) pseudo-first-order and (d) pseudo-second-order.

The isotherm equations for the Langmuir and Freundlich models have been tested in this study. The linear equations were used to fit the Cd^2+^ adsorption process on MS-50 presented in Fig. 8a and b, and the isotherm parameters are shown in Table 5. The Langmuir isotherm assumes a homogeneous adsorption process.^13^ The Langmuir equation is calculated as,3
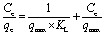
*C*_e_ and *q*_e_ are the equilibrium concentration and amount of adsorbate at equilibrium (mg g^−1^), *q*_max_ and *K*_L_ are the maximum adsorption capacity (mg g^−1^) and equilibrium constant or Langmuir constant of adsorption, respectively. The Langmuir adsorption isotherm was obtained by plotting 1/*C*_e_*versus* 1/*q*_e_.

Parameters of the Langmuir, Freundlich, pseudo-first-order and pseudo-second-order models for adsorption of Cd^2+^ onto MS-50

**Table:** 

Langmuir			Freundlich		
*q*_m_ (mg g^−1^)	*K*_L_	*R* ^2^	*K*_F_ (mg g^−1^)	*n*	*R* ^2^
103.10	0.07	0.90	12.22	1.97	0.83

**Table:** 

Pseudo-first-order			Pseudo-second-order		
*q*_e_ (mg g^−1^)	*k*_1_ (min^−1^)	*R* ^2^	*q*_e_ (mg g^−1^)	*k*_2_ (min^−1^)	*R* ^2^
0.59	0.005	0.93	8.26	0.12	0.99

The Freundlich adsorption isotherm is an empirical equation employed to describe heterogeneous systems.^50^ The Langmuir equation is calculated as,4
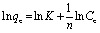
*C*_e_ and *q*_e_ are the equilibrium concentration and amount of adsorbate at equilibrium (mg g^−1^). *K*_F_ and 1/*n* are the Freundlich constant and heterogeneity factors of adsorption, respectively; *n* is a deviation from the linearity of adsorption. Freundlich adsorption isotherms were obtained by plotting log *q*_e_*versus* log *C*_e_.

Results show that the Langmuir model is better than the Freundlich model in simulating the adsorption experiments. The adsorption isotherm of MSME-A suggests that the adsorption process is homogeneous. The maximum adsorption capacities for Cd^2+^ were 103.10 mg g^−1^. During the adsorption process, several parameters are related to the state of the adsorbent and the physicochemical state in which adsorption affects the kinetic reactions. The pseudo-first-order and pseudo-second-order kinetics models were used to determine that the adsorbent took up Cd^2+^. The linear equation pseudo-first-order is calculated as,^51^

5 ln(*q*_e_ − *q*_*t*_) = ln *q*_e_ − *k*_1_*t*

*q*_e_ and *q*_*t*_ are the amount adsorbed at equilibrium time (mg g^−1^) and any time *t*, respectively. *k*_1_ is the rate constant for first-order adsorption. The pseudo-first-order was obtained by plotting time *versus* log(*q*_e_ − *q*_*t*_).

The linear equation of pseudo-second-order is calculated as,6
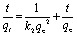
*q*_e_ and *q*_*t*_ are the amount adsorbed at equilibrium time (mg g^−1^) and any time *t*, respectively. *k*_2_ is the rate constant for second-order adsorption. The pseudo-second-order was obtained by plotting time *versus t*/*q*_*t*_.

The plots of the pseudo-first-order and pseudo-second-order for different initial Cd^2+^ concentrations are shown in Fig. 8c and d. The adsorption kinetics data and the predicted model parameters are shown in Table 5. Among these parameters, the correlation coefficient (*R*^2^) and the agreement between the calculated and experimental values of *q* are the most important. It is used to confirm that the models can be used. The excellent agreement between the calculated *q*_e_ and the theoretical *q*_e_ and the high values of *R*^2^ (*R*^2^ = 0.99) show that the pseudo-second-order kinetic model gives a good description of this adsorption process. This means that the limiting step for removal may be a chemisorption process.

A comparison between the adsorption capacities (*q*_m_) values of different adsorbents reported in the literature and that of MS-50 for adsorption of Cd^2+^ can be seen in Table 6. It may be seen that *q*_m_ values differ widely for different adsorbents. Comparison of *q*_m_ values also shows that MS-50 exhibited a reasonable capacity for adsorption of Cd^2+^ from aqueous solutions. Mesoporous silica Cd adsorption can be done repeatedly.

**Table:** Adsorption capacity of different adsorbents for Cd^2+^ adsorption adsorbent

Adsorbent	Adsorption capacity (mg g^−1^)	Ref.
Orange peel Fe_2_O_3_	71.43	52
*Prunus avium* leaves	45.45	53
Kiwi cortex	15.9	54
Unmodified rice straw	13.9	55
Coffee grounds	15.65	56
Activated carbon–NaOH	100	57
Agave bagasse	14	58
Padina gymnospora	99.85	59
Silica	33.33	60
MS-50	103.10	This study

## 4. Conclusion

The stability of mesoporous silica using ricinoleic methyl ester as a template with the addition of HCl has been done. FT-IR results of all silica material products in all reaction conditions showed the formation of Si–OH, Si–O–Si, and Si–O. The XRD analysis results showed that all silica are amorphous. SEM images show that the mesoporous silica was spherical. The results of the nitrogen adsorption characterization show that MS-50 has a large surface area and porosity, resulting in good mesoporous silica. According to the final model equation for the coded factors, the pH, adsorbent dose, Cd^2+^ concentration, and contact time (in that order) had the most significant effect on the efficiency of the process. About 86.63% of the Cd^2+^ was taken out under the best conditions. Based on the isotherm studies, the Langmuir model was the best fit for the equilibrium data, and the maximum adsorption capacity was 103.10 mg g^−1^.

## Conflicts of interest

The authors state that they have no known conflicting financial or personal interests that might have influenced the work presented in this study.

## Supplementary Material
